# A State-of-the-Art Review of Dementia Caregivers with Cardiometabolic Diseases: Evidence and Future Directions

**DOI:** 10.1093/geroni/igaf122.3693

**Published:** 2025-12-31

**Authors:** Jin Su, Shicheng Xu, Jiaming Xiong, Xiang Qi, Yaguang Zheng, Jing Wang, Weiyu Mao, Bei Wu

**Affiliations:** New York University, New York, New York, United States; New York University, New York, New York, United States; The Hong Kong Polytechnic University, Hong Kong, Hong Kong, China (People’s Republic); New York University, New York, New York, United States; New York University, New York, New York, United States; University of New Hampshire, Durham, New Hampshire, United States; University of Nevada, Reno, Reno, Nevada, United States; NYU Shanghai, Shanghai, China (People’s Republic)

## Abstract

Informal caregivers of people with dementia face an increased risk of cardiometabolic diseases due to demanding caregiving responsibilities. Notably, 80% of these caregivers experience at least one chronic condition, yet they often prioritize caregiving over their health. Despite this vulnerability, evidence on interventions targeting dementia caregivers with cardiometabolic diseases remains limited. This State-of-the-Art review aims to synthesize existing knowledge on dementia caregivers with cardiometabolic diseases, identify research challenges, and highlight future directions. A systematic search of Medline, CINAHL, Embase, and PsycINFO identified 6,657 articles published between Jan 1996 and May 2025. Inclusion criteria focused on studies of dementia caregivers with at least one cardiometabolic or chronic disease, excluding non-English studies, conference abstracts, reviews, and editorials. Five studies remained in the final review: four survey studies (three cross-sectional and one longitudinal) and one intervention proposal study. Three themes emerged from the studies: (1) the synergistic effects of multiple cardiometabolic diseases on stress responses and metabolic risks among dementia caregivers; (2) the need for multicomponent interventions addressing stress and lifestyle in dementia caregivers with cardiometabolic diseases, and (3) the underexplored potential of mobile health technology to support self-management among dementia caregivers with cardiometabolic diseases. The review highlights the scarcity of research on this population, likely due to methodological challenges related to their compounded health risks and complex support needs. Future studies should explore innovative and flexible methods to engage dementia caregivers with cardiometabolic diseases, particularly those from diverse cultural and socioeconomic backgrounds, and develop tailored strategies.

